# Lectin from *Vatairea macrocarpa* (Benth.) Ducke Exhibits Selective Cytotoxicity and Angiogenesis Inhibition in Lung Cancer Cells

**DOI:** 10.1021/acsomega.5c08096

**Published:** 2025-10-08

**Authors:** Adrielle Rodrigues Costa, Renato Rodrigues Roma, Abel Vieira de Melo Bisneto, Felipe Eduardo Alves De Paiva, Jefferson Hollanda Véras, Juliana Santana De Curcio, Lívia Do Carmo Silva, Lee Chen-Chen, Cléver Gomes Cardoso, Elisângela de Paula Silveira-Lacerda, Claudener Souza Teixeira

**Affiliations:** † Medicine Department, 423875 Regional University of Cariri - URCA, Crato, CE 63048-080, Brazil; ‡ Center for Agricultural Sciences and Biodiversity, Federal University of Cariri, Crato, CE 74605-220, Brazil; § Laboratory of Radiobiology and Mutagenesis, Department of Genetics, Institute of Biological Sciences, Federal University of Goiás, 74690-900 Goiânia, Brazil; ∥ Laboratory of Molecular Genetics and Cytogenetics, Department of Genetics, Institute of Biological Sciences, Federal University of Goiás, 74690-900 Goiânia, Brazil

## Abstract

Angiogenesis plays a vital role in tumor development, and its inhibition, along with selective cytotoxicity, represents a promising strategy for cancer treatment. Lectins, carbohydrate-binding proteins, have demonstrated dual potential in blocking angiogenesis and selectively targeting tumor cells. This study investigates the antiangiogenic and cytotoxic properties of *Vatairea macrocarpa* lectin (VML) through the chorioallantoic membrane (CAM) assay and tests on normal VERO cells and tumor cell lines A549, SH-SY5Y, S180, and B16-F10. VML exhibited selective cytotoxicity exclusively against A549 lung carcinoma cells, with an IC_50_ of 97.21 μg/mL, showing no significant toxicity to other lines. In the CAM assay, VML significantly inhibited neovascularization triggered by A549 cells, reaching 70.38% inhibition at 100 μg/mL. Immunohistochemical analyses confirmed the suppression of angiogenesis by showing decreased expression of VEGF and TGF-β. Histological assessments also revealed reductions in new vessel formation, inflammatory cell infiltration, fibroblast presence, and membrane thickening. These results highlight VML’s dual role in inhibiting angiogenesis and exerting selective cytotoxicity, likely due to its specific interaction with tumor-associated carbohydrates. Consequently, VML emerges as a potential candidate for targeted cancer therapy or as a complementary therapeutic agent. Further research is necessary to fully understand the molecular mechanisms underlying its antitumor activity.

## Graphical abstract

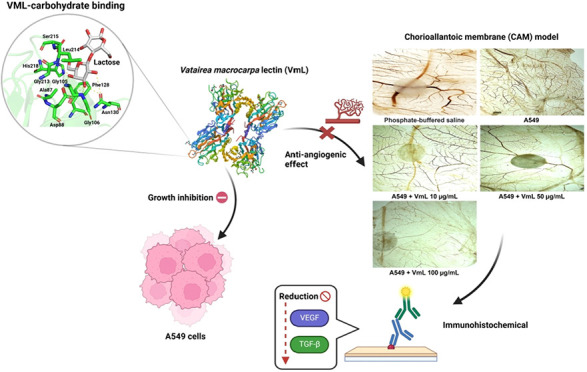

## Introduction

According to the World Health Organization (WHO), cancer is currently the second leading cause of death worldwide, surpassed only by cardiovascular diseases.^1,2^ The global burden of cancer is steadily increasing, with projections indicating that by 2030, cancer-related deaths in the Americas may reach approximately 2.1 million, and one in every six deaths worldwide could be attributed to cancer.^1^

A comprehensive understanding of the mechanisms underlying cancer progression is essential for the development of more targeted and individualized therapeutic strategies. For instance, integrating angiogenic inhibition with selective cytotoxicity has emerged as a promising approach to enhance treatment efficacy while minimizing harm to healthy tissues.^3–6^

Angiogenesis, defined as the formation of new blood vessels, plays a crucial role in tumor progression and has been extensively investigated as a therapeutic target in cancer treatment. Solid tumors depend on an established blood supply to sustain rapid growth and facilitate metastasis. To meet these demands, cancer cells secrete a variety of growth factors, such as VEGF and TGF-β, along with other angiogenic proteins that stimulate neovascularization, ensuring a continuous supply of oxygen and nutrients to the tumor.^7,8^ Consequently, antiangiogenic agents targeting these vascular growth factors and key molecules involved in neovascularization have been employed to restrict the tumors’ blood supply, thereby inhibiting tumor growth and metastasis.^9,10^

Another promising approach in cancer research involves developing agents that target specific glycosylated structures on cell surfaces. Cancer cells display aberrant glycosylation patterns throughout tumorigenesis, affecting both O-linked and N-linked glycans. These modifications, recognized as hallmarks of tumorigenesis, are closely associated with tumor progression and metastasis.^11^ Specific altered glycans have been identified as tumor-associated antigens, serving as valuable biomarkers for cancer diagnosis and prognosis.^12^ Notably, many tumor cells overexpress O-linked glyco-epitopes, such as the Thomsen-Friedenreich antigen (TF or T), Thomsen-nouvelle antigen (Tn), sialyl-Tn (sTn), sialyl-Lewis x, and sialyl-Lewis∧um (sLex/Sleum). Collectively, these are referred to as tumor-associated carbohydrates (TACs), making them attractive targets for cancer immunotherapy.^13,14^

In this context, lectins, proteins that recognize and interact with both free and conjugated carbohydrates on cell surfaces, have gained significant attention due to their specific carbohydrate-binding domains. They bind reversibly to membrane carbohydrates via noncovalent interactions without altering the carbohydrate structures, thereby enabling them to recognize a diverse array of glycan profiles.^15–17^ Moreover, these lectin–carbohydrate interactions can trigger various cellular responses, such as cytotoxic and anticancer effects,^18^ pro-inflammatory responses,^19^ angiogenic modulation,^15^ and antimicrobial activity.^20^

In plants, lectins are present in various families, with the Fabaceae being particularly enriched in these proteins. A notable example is the lectin derived from *Vatairea macrocarpa* (Benth.) Ducke (VML). Extracted from the seeds of this species, VML exhibits a high affinity for galactose/N-acetylgalactosamine (Gal/GalNAc) carbohydrates, carbohydrates that are characteristic of tumor-associated carbohydrates (TACs) found on the surface of cancer cells.^20,21^ The lectin has several applications described in the literature, including macrophage activation,^22^ renal effects induced,^23^ pro-inflammatory activity,^24^ microbiological activity,^20^ *in vitro* antiproliferative effect on leukemia cells^25^ and antiangiogenic activity.^15^

Therefore, the present study investigates the *in vitro* cytotoxic potential of VML on both tumor and normal cells and its capacity to inhibit angiogenesis induced by A549 cells *in vivo* using the chick embryo chorioallantoic membrane (CAM) assay. Additionally, immunohistochemical analyses were performed to assess the expression of VEGF and TGF-β.

## Materials and Methods

### Obtaining License and Botanical Material Collection

A request for a license to collect botanical material was submitted to the environmental agency SISGEN (National System for the Management of Genetic Heritage) under registration number ID: AF8E1DD. After obtaining the necessary licenses, seeds of the species were collected in the municipality of Chapadinha - MA, Brazil, at coordinates 03° 44′ 05.9″ S and 43° 19′ 02.0″ W. The voucher specimen was deposited in the Herbarium Caririense Dárdano de Andrade Lima at the Universidade Reguinal do Cariri (URCA) with the voucher number 15.114.

### Purification of *Vatairea macrocarpa* Lectin (VML)

A soluble protein extract was initially prepared from the seeds of *Vatairea macrocarpa* to purify the lectins. The extracts were precipitated with 60% ammonium sulfate, and the resulting precipitate was applied to a guar gum (2 × 10 cm) affinity chromatography column as previously described by Santos et al.^20^ The purity of the lyophilized lectin samples was confirmed by SDS-PAGE 12.5% electrophoresis.

### Cytotoxicity

#### Cell Line Culture

Tumor cell lines used: A549 – Lung carcinoma (ATCC CCL-185), SH-SY5Y – Neuroblastoma (ATCC CRL-2266), S180 – Sarcoma (TCC CCL TIB-66), B16–F10 – Murine melanoma (ATCC CRL-6475), and normal cell line: VERO – kidney epithelial cell from *Cercopithecus aethiops* (ATCC CCL-81). S180 cells were cultured in Roswell Park Memorial Institute medium - RPMI1640 (Gibco), and the others were cultured in Dulbecco’s Modified Eagle’s Medium - DMEM (Sigma Chemical Co., MO), supplemented with 10% fetal bovine serum, 100 μg/mL penicillin, and 100 μg/mL streptomycin. The cultures were incubated in a humidified incubator (NUAIRE model TS Autoflow) at 37 °C with 5% CO_2_.^2^

#### Cell Viability Assay (MTT Assay)

The cytotoxicity of the lectins was evaluated using an MTT colorimetric assay with cancer cells A549, S180, SH-SY5Y, B16–F10, and VERO. Cells (10 × 10^4^) were plated in 96-well culture plates and treated with different concentrations of VML (6.25–200 μg/mL) for 48 h. After treatment, 20 μL of MTT (5 mg/mL) was added to each well, and the plates were incubated at 37 °C for an additional 3 h. The culture medium was discarded, and the cells were washed with PBS. Formazan crystals were dissolved in 70 μL of DMSO, and absorbance was measured at 545 nm using a microplate reader. Cell viability was calculated as follows

viability(%)=100−(treatment
absorbance/control absorbance)

inhibitionrate(%)=[(controlabsorbance−sampleabsorbance)/controlabsorbance]×100

Additionally, an inhibition assay was performed to investigate whether the carbohydrate-binding ability of VML plays a role in cytotoxicity against A549 cancer cells. VML (IC_50_) was incubated with lactose (0.1 M) at 37 °C for 30 min before evaluating cytotoxic activity. The A549 cells were then treated with VML (IC_50_ – 97.21 μg/mL), VML combined with lactose (0.1 M), denatured VML (heated for 1 h at 100 °C), and lactose alone. These treatments were incubated at 37 °C with 5% CO_2_ for 48 h, followed by the MTT assay as described above.

### Evaluation of the Antiangiogenic Response with A549 Cells

#### Chick Embryo Chorioallantoic Membrane (CAM) Assay

In this study, fertilized chicken eggs (*Gallus gallus domesticus*) were obtained from the Toca dos Lobos farm (Goiás, Brazil). The antiangiogenic response of VML was evaluated using a CAM assay, based on the protocol described by Auerbach et al.^26^ with some modifications.^27^ Twenty-five fertilized chicken eggs were placed for incubation horizontally at 37 °C with a relative humidity of 80% in a BOD chamber (Model SL224). On the seventh day of incubation, a circular opening was made in the widest part of the egg, and the shell membrane was removed to assess the normal development of the CAM. The eggshells were properly sealed, and the eggs were placed back in the incubator.

On the twelfth day of incubation, the eggs were divided into five treatment groups (five eggs/group): (1) Inducing control group, containing only the presence of A549 cells, (2) Negative control group, (3) A549 + 10 μg/mL VML group, (4) A549 + 50 μg/mL VML group, (5) A549 + 100 μg/mL VML group. Twenty μL of each concentration was pipetted onto a sterile paper filter disc previously placed on the chorioallantoic membrane of each egg. After 72 h of treatment, the embryos were euthanized and the angiogenic response was analyzed using images captured with a digital camera (Nikon Coolpix L810 16.1 megapixels).

Quantification of vascularization in percentage was performed using ImageJ software (version 1.51j8, National Institutes of Health, Bethesda, MD). AngioQuant software (version 1.33, Institute of Signal Processing, Finland) was used to determine the length and caliber of blood vessels, as well as the number of vascular complexes and junctions.

#### Histological Analysis of the CAM

The CAM was immersed in a formaldehyde solution (10%; pH 6.9) for 24 h. It was then subjected to a gradual dehydration process in ethanol, followed by diafanization in xylene, and finally embedded in paraffin. The samples were cut into 5 μm slices using a semiautomatic microtome (Leica model RM2245) and placed on microscope slides. The sections were deparaffinized with xylene and ethanol. The samples were then stained with hematoxylin and eosin (HE) and examined under a light microscope.^28^

Histological analysis followed four distinct parameters for tissue evaluation using a light microscope (Olympus model BH 2) equipped with a 40× objective lens. The parameters included: (I) Neovascularization; (II) Presence of inflammatory elements; (III) Presence of fibroblasts; (IV) Thickening of the chorioallantoic membrane.

The results were visually scored using the following scale: absent (0), slight (1), moderate (2), and intense (3).

#### Immunohistochemical Analysis of the CAM

Sections of 4 μm of the CAM in paraffin underwent a deparaffinization process using xylene and ethanol, followed by washes with phosphate-buffered saline (PBS). Next, the samples were subjected to antigen retrieval in citrate buffer (pH 6) for 45 min. Endogenous peroxidase activity in the samples was inhibited with hydrogen peroxide (H_2_O_2_). All histological samples were incubated with specific antibodies, including antibodies against vascular endothelial growth factor (VEGF; monoclonal mouse IgG sc-53462; Santa Cruz Biotechnology) and transforming growth factor β (TGF-β; rabbit polyclonal IgG sc-7892; Santa Cruz Biotechnology) at a concentration of 1:400. This incubation occurred at 4 °C in a humid chamber overnight. Subsequently, the sections were incubated with a goat antimouse secondary antibody conjugated with peroxidase (1:500; 113-035-003; Jackson ImmunoResearch Laboratories) at room temperature for 3 h. The immunoreactivity was evaluated according to the methodology of Lokman et al.,^29^ using the chromogenic substrate Novocastra DAB (1:50), and the sections were counterstained with hematoxylin. Subsequently, they were analyzed under a light microscope (Leica, model DM2500) with a 40× objective lens.

**Figure 1:**
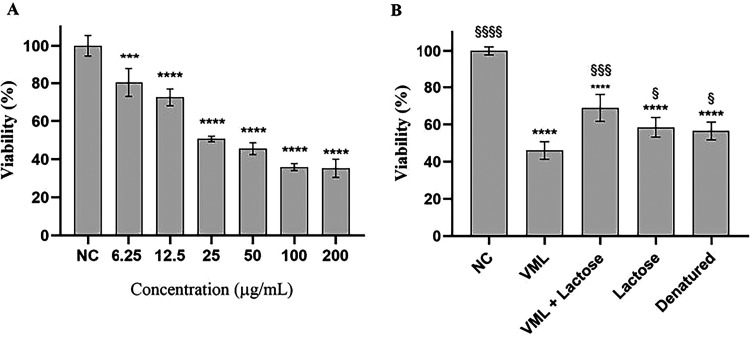
Cytotoxicity of *Vatairea macrocarpa* lectin (VML) in A549 tumor cell line - Lung carcinoma (ATCC CCL-185), after 24 h of incubation. (A) Negative control (NC - PBS) and treatment with different concentrations of active VML (200–6.25 μg/μL). (B) Negative control (NC), native VML (IC_50_ μg/mL), VML combined with lactose, isolated lactose (0.1M), and denatured VML. * Significant difference when compared to the negative control group (**** *p* < 0.0001 and *** *p* < 0.001). § Significantly different when compared to the active VML group (§§§§ *p* < 0.0001, §§§ *p* < 0.001, and § *p* < 0.05).

**Table 1:** Mean Inhibitory Concentration (IC_50_ in μg/mL)

cells	A549	S-180	SH-SY5Y	B16-F10	VERO
VML	97.21	>200	>200	>200	>200

### Statistical Analyses

The results were expressed as mean ± standard error of the mean. Statistical differences between groups were determined using analysis of variance (ANOVA), followed by Dunnett’s or Bonferroni’s multiple comparisons tests when appropriate to detect differences between controls and treatments, using GraphPad Prism software (version 6). The significance level was set at *p* < 0.05. The IC_50_ values (concentration required to inhibit 50%) were calculated to assess cytotoxicity using nonlinear regression with 95% confidence intervals. The mean values obtained for each histological and immunohistochemical parameter were compared using one-way ANOVA, followed by Tukey’s posthoc test.

## Results

### Cytotoxicity

The data presented in Table 1 demonstrated that VML exerted a significant inhibitory effect on A549 tumor cells, with an IC_50_ of 97.21 μg/mL, corresponding to a 73.79% inhibition rate. This response was dose-dependent, as illustrated in Figure 1A, suggesting a high degree of selectivity toward this particular tumor cell line. In contrast, no cytotoxic effects were observed in the other tumor cell lines tested (S180, SH-SY5Y, and B16-F10) or in the normal cell line VERO, even at concentrations as high as 200 μg/mL.

To further investigate the role of VML carbohydrate-binding activity in its cytotoxic effects, A549 cells were cotreated with the IC50 concentration of VML (97.21 μg/mL) and lactose (0.1 M). This cotreatment resulted in a reduction of VML’s cytotoxicity, as shown in Figure 1B. Additionally, the effect of denatured lectin was evaluated, demonstrating that the loss of the native protein structure eliminated its cytotoxic activity. These results indicate that the cytotoxic effect of VML is mediated by its ability to recognize glycans on the surface of A549 cells.

*Vatairea macrocarpa* (VML) lectin, A549 - Lung carcinoma (ATCC CCL-185), S180 - Sarcoma (TCC CCL TIB-66), SH-SY5Y - Neuroblastoma (ATCC CRL-2266), B16–F10 – Murine melanoma (ATCC CRL-6475) and VERO - normal renal cell from *Cercopithecus aethiops* (ATCC CCL-81).

### Macroscopic Antiangiogenic Activity of VML in the CAM Assay

The antiangiogenic potential of VML was evaluated using the CAM assay, in which A549 tumor cells served as the angiogenic stimulus. Figure 2A demonstrates that VML significantly reduced blood vessel formation in the CAM, with a dose-dependent decrease in the percentage of vascularization at all tested concentrations (10, 50, and 100 μg/μL), reaching a 56% inhibition at the highest concentration.

**Figure 2:**
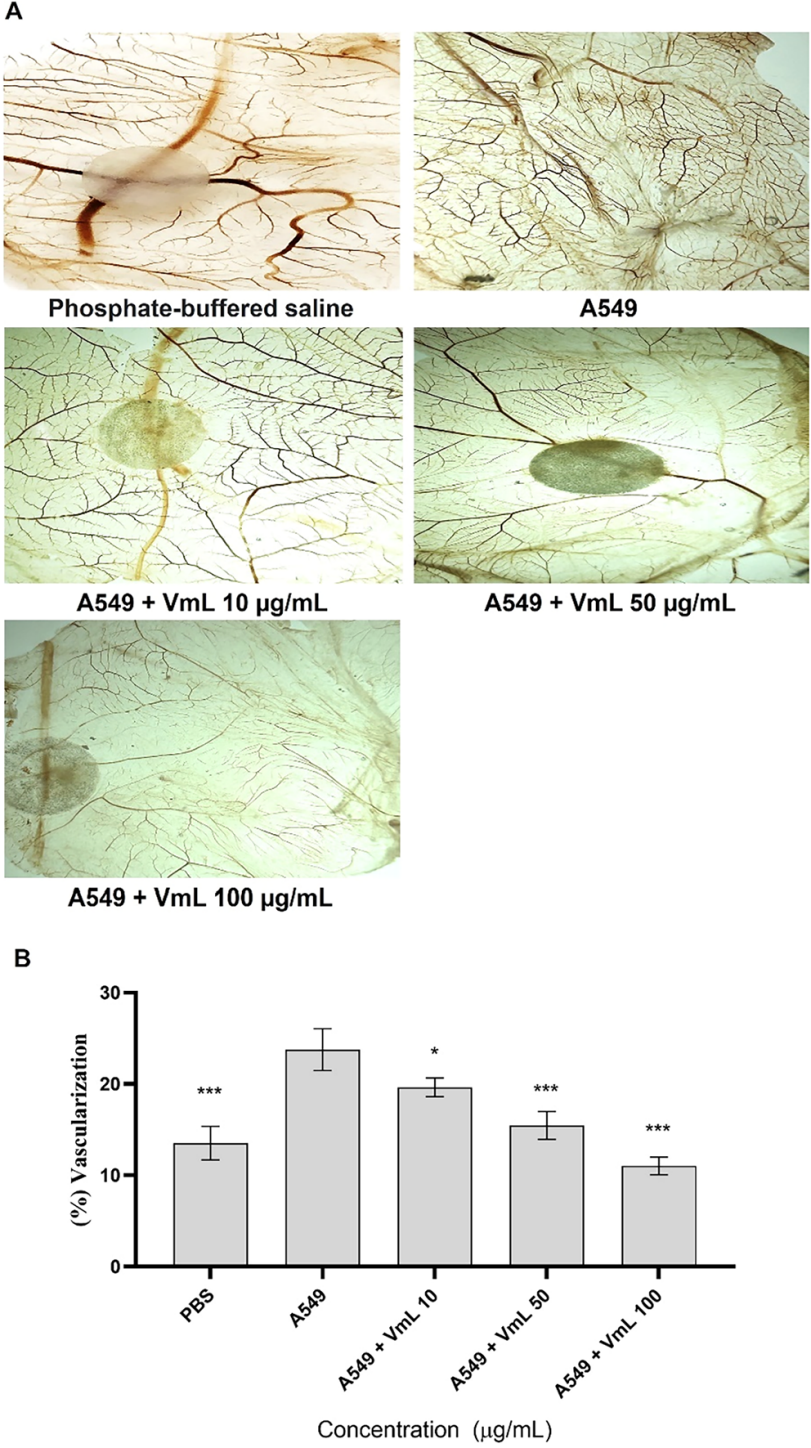
Antiangiogenic activity of *Vatairea macrocarpa* lectin (VML) assessed by the chicken embryo chorioallantoic membrane (CAM) assay. (A) Representative images of different CAMs incubated with A549 after 72 h of treatment with varying concentrations of VML (10, 50, and 100 μg/μL). (B) The average values obtained from each treatment were used to determine vascularization (%). Phosphate-buffered saline (PBS; negative control); Adenocarcinomic human alveolar basal epithelial cells (A549; angiogenesis inducer); VML: *Vatairea macrocarpa* lectin. * Significant difference when compared to the negative control group (***p < 0.001 and *p < 0.05).

To further investigate the mechanism underlying VML antiangiogenic activity, an inhibition test was conducted as shown in Figure 3. The results indicated that both denaturation of the lectin and occupation of its binding site by the carbohydrate abolished its ability to inhibit angiogenesis, suggesting that the lectin specific interaction with carbohydrates is crucial for its biological activity.

**Figure 3:**
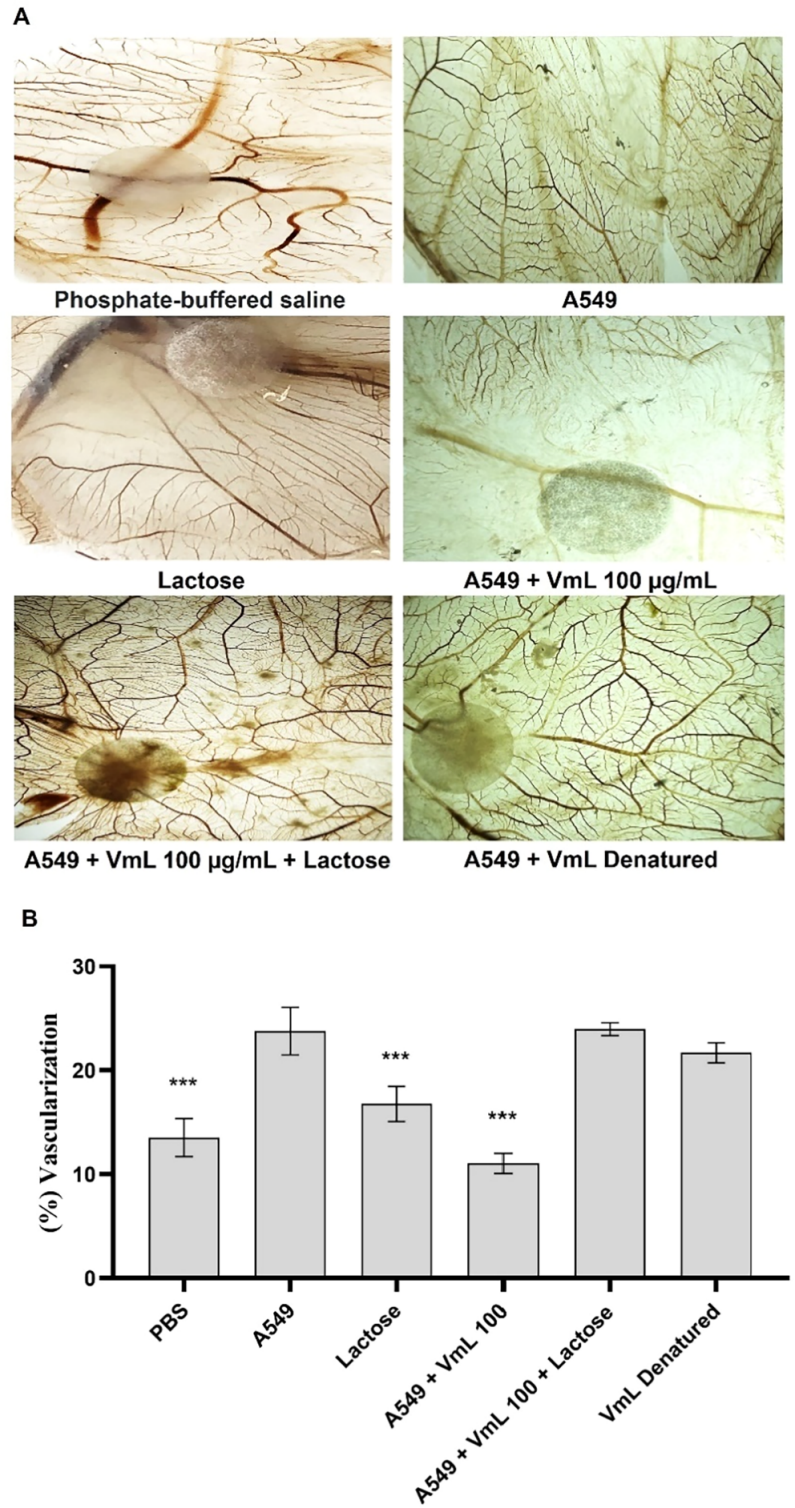
Antiangiogenic activity of *Vatairea macrocarpa* lectin (VML) assessed by the chicken embryo chorioallantoic membrane (CAM) assay, inhibition assay. (A) Representative images of different CAMs incubated with A549 after 72 h of treatment with isolated lactose, VML (100 μg/μL), VML (100 μg/μL) incubated with lactose, and denatured VML. (B) The average values obtained from each treatment were used to determine vascularization (%). Phosphate-buffered saline (PBS; negative control); Adenocarcinomic human alveolar basal epithelial cells (A549; angiogenesis inducer); VML: *Vatairea macrocarpa* lectin. * Significant difference when compared to the negative control group (***p < 0.001).

As presented in Tables 2 and 3, detailed analysis of blood vessel parameters, including length, caliber, and the number of complexes and junctions, revealed a significant concentration-dependent decrease (*p* < 0.05) in most measured parameters following VML treatment, confirming its potent antiangiogenic effects.

**Table 2:** Means ± Standard Deviation of Parameters Analyzed in Chick Embryo Chorioallantoic Membranes (CAM), Treated with Different Concentrations of VML by AngioQuant Software[Table-fn t2fn1]

treatments (μg/mL)	length (pixel)	caliber (pixel)	number of complexes	number of junctions
PBS	343.1 ± 23.2[Table-fn t2fn2]	806.4 ± 101.8[Table-fn t2fn2]	136.3 ± 14.0[Table-fn t2fn2]	105.1 ± 10.3[Table-fn t2fn2]
A549	732.7 ± 33.6	2155.0 ± 217.7	380.1 ± 13.7	256.2 ± 13.3
A549 + VML 10	609.6 ± 140.0[Table-fn t2fn2]	1953.2 ± 167.5[Table-fn t2fn2]	216.0 ± 15.2[Table-fn t2fn2]	197.7 ± 17.2
A549 + VML 50	575.0 ± 120.8[Table-fn t2fn2]	1627.3 ± 206.7[Table-fn t2fn2]	193.7 ± 14.4[Table-fn t2fn2]	107.4 ± 13.0[Table-fn t2fn2]
A549 + VML 100	450.2 ± 123.6[Table-fn t2fn2]	1450.3 ± 150.0[Table-fn t2fn2]	95.5 ± 13.3[Table-fn t2fn2]	79.8 ± 12.1[Table-fn t2fn2]

* Phosphate-buffered saline (PBS; negative control); adenocarcinomic human alveolar basal epithelial cells (A549; angiogenesis inductor); VML: *Vatairea macrocarpa* lectin. ANOVA and Tukey’s posthoc test.

** Significant difference compared to the angiogenesis inductor (*p* < 0.05).

**Table 3:** Means ± Standard Deviation of Parameters Analyzed in Chick Embryo Chorioallantoic Membranes (CAM), Treated with Different Concentrations of VML by AngioQuant Software[Table-fn t3fn1]

treatments (μg/mL)	length (pixel)	caliber (pixel)	number of complexes	number of junctions
PBS	343.1 ± 23.2[Table-fn t3fn2]	806.4 ± 101.8[Table-fn t3fn2]	136.3 ± 14.0[Table-fn t3fn2]	105.1 ± 10.3[Table-fn t3fn2]
A549	702.6 ± 32.2	2135.0 ± 215.1	368.8 ± 17.3	256.2 ± 18.5
lactose	323.3 ± 33.8[Table-fn t3fn2]	869.9 ± 128.3[Table-fn t3fn2]	148.4 ± 15.4[Table-fn t3fn2]	103.0 ± 10.0[Table-fn t3fn2]
A549 + VML 100	425.0 ± 78.5[Table-fn t3fn2]	1325.3 ± 105.0[Table-fn t3fn2]	100.5 ± 10.3[Table-fn t3fn2]	68.5 ± 11.5[Table-fn t3fn2]
A549 + VML 100 + Lactose	682.3 ± 39.5	2078.5 ± 217.7	359.9 ± 19.7	246.6 ± 19.8
A549 + VML denatured	607.7 ± 27.3	1985.8 ± 217.7	337.7 ± 15.2	238.5 ± 15.7

* Phosphate-buffered saline (PBS; negative control); adenocarcinomic human alveolar basal epithelial cells (A549; angiogenesis inducer); Lactose (VML binding carbohydrate); VML: *Vatairea macrocarpa* lectin. ANOVA and Tukey’s posthoc test.

** Significant difference compared to the angiogenesis inductor (*p* < 0.05).

### Histological Analysis of the CAM

As summarized in Table 4, VML treatment resulted in a significant reduction in all evaluated parameters, including neovascularization, the presence of inflammatory cells, fibroblasts, and thickening of the chorioallantoic membrane. This inhibitory effect was more pronounced at higher VML concentrations (50 and 100 μg/mL), demonstrating a dose-dependent response, indicating that as the concentration of lectins increases, their action becomes more effective. These findings collectively suggest that VML exerts a potent antiangiogenic effect. Furthermore, Table 5 presents data related to the inhibition of this effect, showing no significant results for the same parameters when the lectin is denatured or incubated with lactose.

**Table 4:** Histological Analysis of Chick Embryo Chorioallantoic Membranes (CAM)[Table-fn t4fn1],[Table-fn t4fn2]

treatments (μg/mL)	neovascularization	presence of inflammatory cells	presence of fibroblastos	thickening in chorioallantoic membrane
PBS	1.0 ± 0.5[Table-fn t4fn3]	1.5 ± 0.3[Table-fn t4fn3]	1.2 ± 0.3[Table-fn t4fn3]	1.0 ± 0.5[Table-fn t4fn3]
A549	2.0 ± 0.3	3.0 ± 0.8	2.5 ± 0.6	3.0 ± 0.5
A549 + VML 10	3.0 ± 0.8	3.0 ± 0.5	2.5 ± 0.5	3.0 ± 0.5
A549 + VML 50	2.0 ± 0.5	2.5 ± 0.5	2.5 ± 0.4	2.0 ± 0.6[Table-fn t4fn3]
A549 + VML 100	1.0 ± 0.1[Table-fn t4fn3]	1.5 ± 0.5[Table-fn t4fn3]	1.5 ± 0.5[Table-fn t4fn3]	1.2 ± 0.3[Table-fn t4fn3]

* Means ± standard deviation of histological parameters classified at a scale of 0-3.

** Phosphate-buffered saline (PBS; negative control); adenocarcinomic human alveolar basal epithelial cells (A549; angiogenesis inducer); VML: Vatairea macrocarpa lectin. ANOVA and Tukey’s posthoc test.

*** Significant difference compared to the angiogenesis inductor (*p* < 0.05).

**Table 5:** Histological Analysis of Chick Embryo Chorioallantoic Membranes (CAM)[Table-fn t5fn1],[Table-fn t5fn2]

treatments (μg/mL)	neovascularization	presence of inflammatory cells	presence of fibroblasts	thickening in chorioallantoic membrane
PBS	1.0 ± 0.5[Table-fn t5fn3]	1.5 ± 0.3[Table-fn t5fn3]	1.2 ± 0.3[Table-fn t5fn3]	1.0 ± 0.5[Table-fn t5fn3]
A549	2.0 ± 0.3	3.0 ± 0.8	2.5 ± 0.6	3.0 ± 0.5
lactose	1.3 ± 0.4[Table-fn t5fn3]	1.0 ± 0.5[Table-fn t5fn3]	1.0 ± 0.5[Table-fn t5fn3]	0.7 ± 0.3[Table-fn t5fn3]
A549 + VML 100	1.0 ± 0.1[Table-fn t5fn3]	1.5 ± 0.5[Table-fn t5fn3]	1.5 ± 0.5[Table-fn t5fn3]	1.2 ± 0.3[Table-fn t5fn3]
A549 + VML 100 Denatured	2.0 ± 0.8	2.5 ± 0.6	2.0 ± 0.5	3.0 ± 0.8
A549 + VML 100 + Lactose	2.0 ± 0.5	2.0 ± 0.3	2.0 ± 0.5	2.5 ± 0.5

* Means ± standard deviation of histological parameters classified at a scale of 0–3.

** Phosphate-buffered saline (PBS; negative control); adenocarcinomic human alveolar basal epithelial cells (A549; angiogenesis inducer); VML: *Vatairea macrocarpa* lectin. ANOVA and Tukey’s posthoc test.

*** Significant difference compared to the angiogenesis inductor (*p* < 0.05).

### Immunohistochemistry

Figure 4 shows a significant reduction in the expression of VEGF (*p* < 0.05) and TGF-β (*p* < 0.001) in the CAM following treatment of A549 tumor cells with VML lectin. This reduction was particularly evident at the highest concentration of VML (100 μg/mL), showing reductions of 71.07% in VEGF and 65.97% TGF-β, when compared to A549 treatment alone.

**Figure 4:**
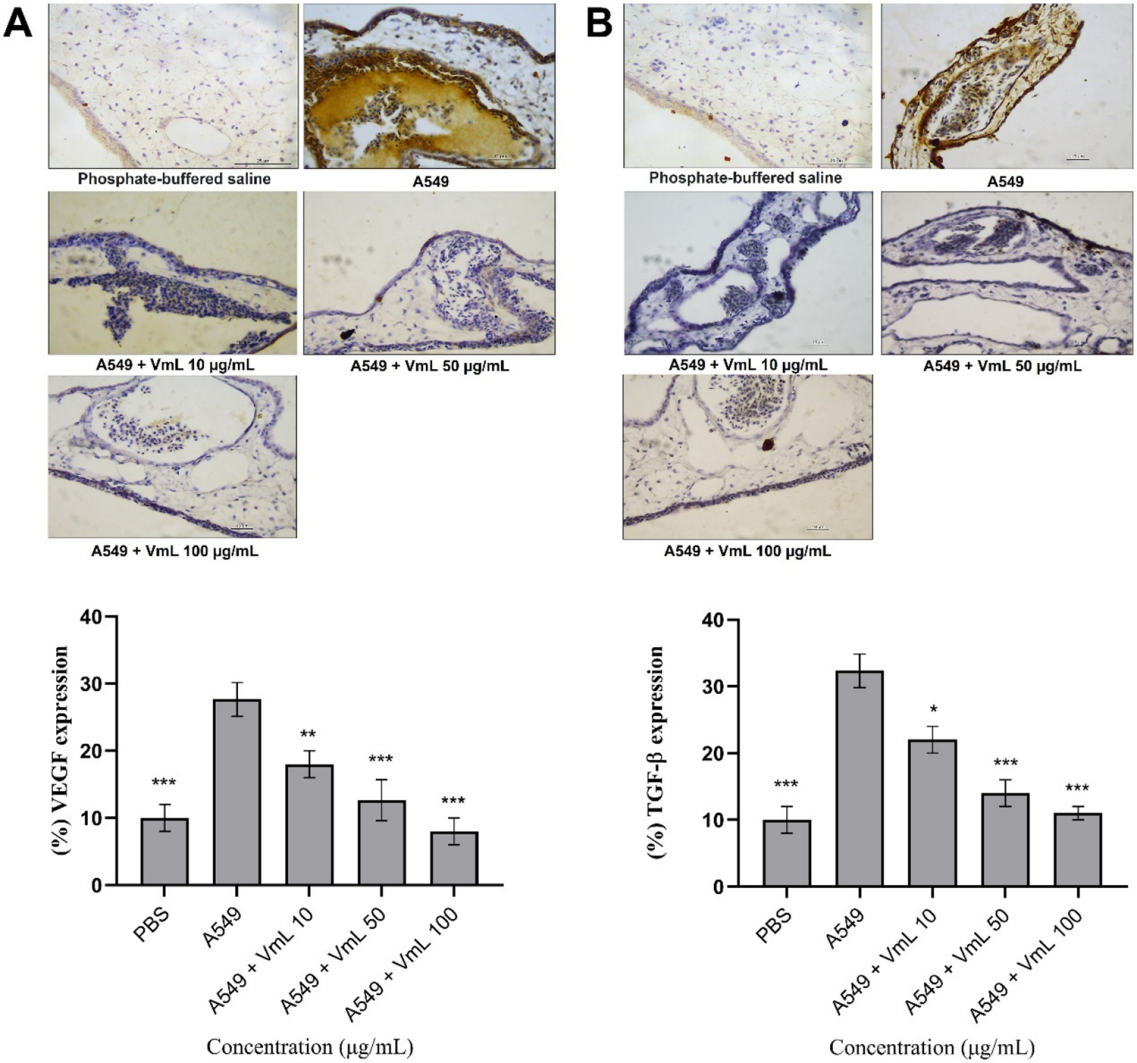
Immunohistochemical analysis of angiogenic factors in the chorioallantoic membrane (CAM) of chicken embryos. (A) Immunological expression of vascular endothelial growth factor (VEGF). (B) Immunological expression of transforming growth factor β (TGF-β). The averages of the values from each treatment were used to calculate relative expression (%). * Significant difference compared to the angiogenesis inducer A549 (***p < 0.001, **p < 0.01 and *p < 0.05).

The results in Figure 5 demonstrated that both denaturation of the lectin and occupation of its carbohydrate-binding site by lactose affected the potential of VML in reducing VEGF or TGF-β expression levels.

**Figure 5:**
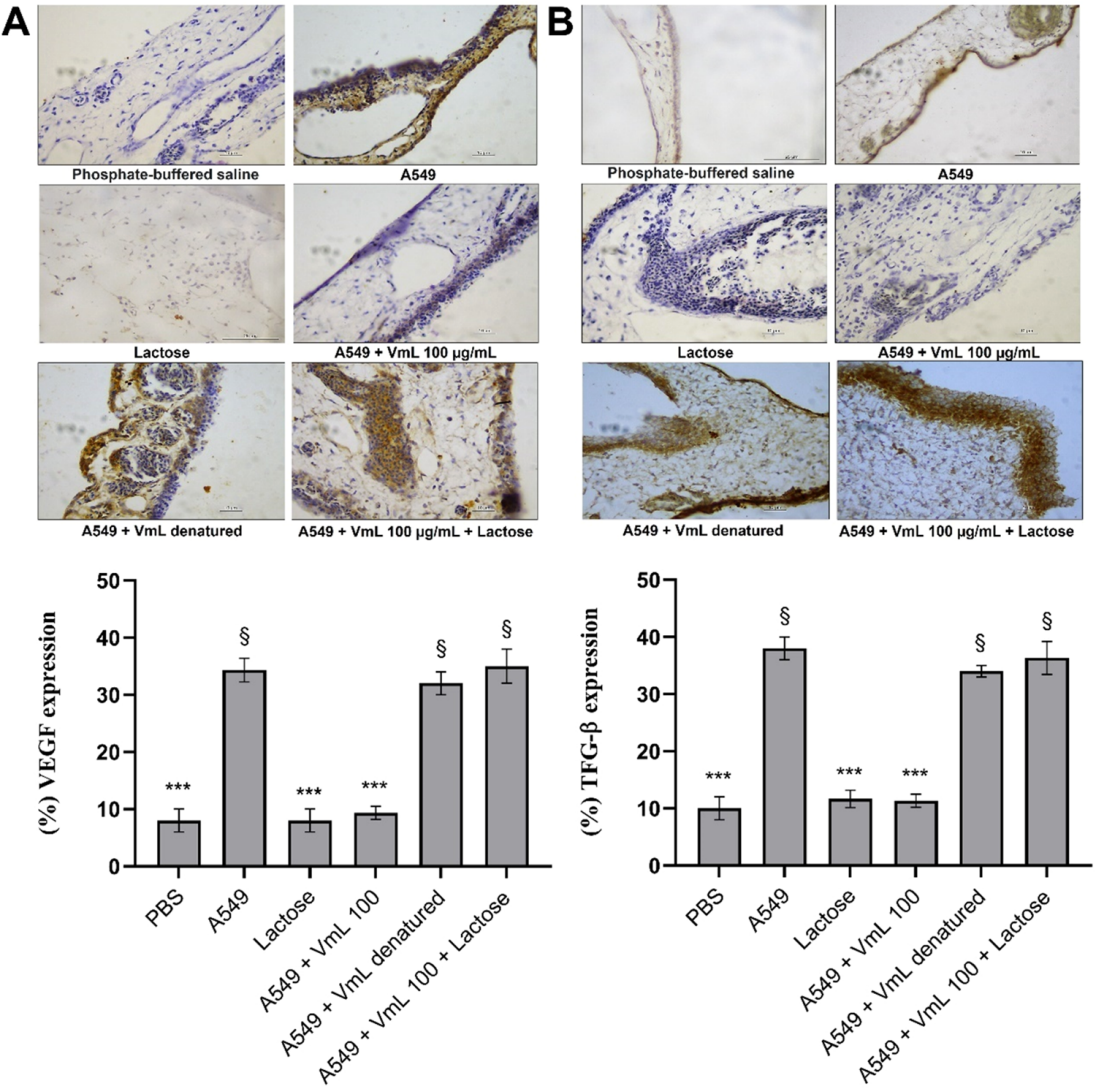
Immunohistochemical analysis of angiogenic factors in the chorioallantoic membrane (CAM) of chicken embryos, inhibition assay. (A) Immunological expression of vascular endothelial growth factor (VEGF). (B) Immunological expression of transforming growth factor β (TGF-β). Representative images of CAM incubated with isolated A549 after 72 h of treatment with isolated lactose, A549 + active VML (100 μg/μL), A549 + denatured, and A549 + VML (100 μg/μL) incubated with lactose. * Significant difference compared to the angiogenesis inducer A549 (*** *p* < 0.001). § Significant difference compared to the lactose group (§ *p* < 0.05).

## Discussion

The selective interactions between lectins and carbohydrates can induce cellular responses that lead to a variety of biological activities, including antitumor and cytotoxic activities.^30,31^ The results revealed that VML lectin exhibited a significant cytotoxic effect specifically against A549 cells, when compared to other tumor lines in our study. In contrast, nontumor cell lines (VERO) remained largely unaffected by the lectin treatment. This selectivity suggests that VML may have a high affinity for specific carbohydrate structures found on altered glyco-epitopes, commonly referred to as tumor-associated carbohydrates (TACs),^14^ present in these tumor cells. This specific interaction is likely to be a key factor contributing to VML cytotoxic activity and its ability to inhibit tumor progression in A549 cells.^14,11,12^

These findings indicate a preferential affinity of VML for cancer cells,^32^ especially lung cancer cells, as other tumor cell lines did not show significant cytotoxic responses. This selective affinity may be attributed to factors such as cell doubling time, cell density, and the distinct growth characteristics of each cell line.^33^ In the case of A549 cells, which have a high proliferation rate, they possess a surface rich in carbohydrates that can interact more intensively with the lectins, resulting in a more pronounced response to the treatment.^33^ Additionally, the interaction between the lectin and tumor cells can be influenced by stimuli from the microenvironment, such as growth factors and specific receptors,^27^ which may be more abundant or more active in A549 cells compared to other tumor cell lines.

These findings are supported by Costa et al.,^25^ who reported that VML demonstrated significant cytotoxicity against the leukemic cell lines HL-60 and KG1, with IC_50_ values of 3.5 μg/mL and 18.6 μg/mL, respectively, while no significant cytotoxicity was observed in the nontumor cell line HaCaT. These results further emphasize the selectivity of the lectin cytotoxic activity, indicating that VML may specifically target certain types of cancer cells.

The investigation conducted by Véras et al.,^15^ employing the CometChip assay in healthy lymphocytes, provides additional evidence supporting the low cytotoxic profile of VML at concentrations comparable to those utilized in the present study. The findings demonstrated that VML concentrations below 8 μM did not elicit genotoxic or cytotoxic effects in nontumor cells. Moreover, the correlation analysis between genotoxicity and cytotoxicity parameters revealed that treatment with VML at 0.5 and 2 μM resulted in cell viability rates of 84% and 74%, respectively.

To further elucidate the role of the lectin’s carbohydrate recognition domain (CRD) in its cytotoxic activity, additional inhibition assays were performed. These experiments indicated that preincubation of VML with lactose significantly attenuated its cytotoxic effects. Furthermore, denaturation of the lectin abolished its cytotoxic activity, confirming that the native conformation of the lectin is essential for its biological function.^15,23,32–35^

The presence of structures like Tn antigens (GalNAcα1-O-Ser/Thr) and the expression of galactose residues on these cells makes them potential targets for VML binding. The lectin specific affinity for galactose and N-acetylgalactosamine residues suggests that it can interact with these glycans which are displayed on the surface of A549 cells. This interaction may influence various biological processes, including cell adhesion, signaling pathways, and inflammatory responses associated with lung cancer (tumor progression of this cell line), as reported by.^36,37^

Angiogenesis is a fundamental process involved in several physiological events, including embryonic development, wound repair, and ocular neovascularization.^38^ In the context of cancer, however, angiogenesis is particularly significant as it is essential for tumor growth and metastasis. The formation of new blood vessels provides tumors with the necessary nutrients and oxygen to sustain their growth and promote their spread to distant sites.^7^ This process is regulated by a complex interplay of various cell types and signaling molecules. Pro-inflammatory cytokines and pro-angiogenic growth factors, such as TGF-β, TNF-α, and VEGF, stimulate endothelial cell proliferation, leading to the formation of new blood vessels. This neovascularization network supports tumor growth and progression.^7,39^

In order to better understand the lectin role in the antiangiogenic process, our experiments demonstrated that VML effectively inhibited A549 cell-induced vessel formation in a dose-dependent manner. However, data from Véras et al.,^15^ showed that VML, when tested alone at concentrations of 0.5, 2, and 8 μM, promoted significant neovascularization in a CAM model, suggesting an intrinsic pro-angiogenic activity of the lectin. These findings indicate that while VML exhibits pro-angiogenic properties (alone), its activity can be modulated in the context of tumor angiogenesis, where it can inhibit vessel formation induced by A549 cells. This dual nature of VML highlights its potential as a therapeutic target for developing novel antiangiogenic therapies aimed at specifically blocking tumor angiogenesis.

Our histological analysis of membranes treated with VML revealed a significant decrease in all evaluated parameters, supporting the antiangiogenic effect observed in the chorioallantoic membrane (CAM) model. Furthermore, when assessing the expression of VEGF and TGF-β, key factors involved in angiogenesis and inflammation,^40^ we found that VML significantly downregulated the expression of both factors in the treated CAM, particularly at concentrations of 50 and 100 μg/mL (*p* ≤ 0.001). These results underscore the potential of VML to negatively regulate angiogenesis and the inflammatory response.

The decrease in these two markers (VEGF and TGF-β) in the CAM membrane is directly linked to reduced neovascularization, a crucial process for tumor growth and metastasis, as it supplies tumor cells with essential nutrients and oxygen.^41,42^ Moreover, the downregulation of VEGF and TGF-β is associated with a decrease in the presence of inflammatory cells and fibroblasts, which are key components of the tumor microenvironment and contribute to tumor growth and progression. The reduction of these factors leads to a thinner CAM, reflecting decreased cellular proliferation and tissue remodeling, characteristics commonly observed in tumor environments.^41–43^

Lectins may exert their effects through mechanisms that extend beyond direct cytotoxicity against tumor cells, including antiangiogenic activity mediated by the inhibition of growth factors. Through this action, they contribute to the modulation of the tumor microenvironment by disrupting the formation of new blood vessels and impairing intercellular communication that promotes tumor progression and metastasis.^27^

## Conclusion

This study highlights, the therapeutic potential of the lectin extracted from *Vatairea macrocarpa* (VML) as a selective and multifunctional antitumor agent. VML exhibited pronounced cytotoxic selectivity against A549 lung cancer cells while maintaining low toxicity toward nontumorigenic VERO cells, underscoring its specificity for malignant cells. Notably, VML also demonstrated significant antiangiogenic activity by markedly reducing tumor-induced neovascularization in the chorioallantoic membrane (CAM) model. This effect was supported by immunohistochemical analyses, which revealed a significant downregulation of key angiogenic markers, VEGF and TGF-β, further validating its role in impairing tumor vascular supply.

The findings provide novel evidence that the native structure of VML, particularly its carbohydrate recognition domain, may be crucial for its dual antitumor activity—combining cytotoxicity with disruption of tumor-associated angiogenesis. These results position VML as a promising candidate for further preclinical development, offering a new avenue in the search for targeted and less toxic cancer therapeutics. Future studies exploring its molecular targets and signaling pathways are warranted to elucidate its mechanisms of action.
